# Efficacy of acupuncture for cocaine dependence: a systematic review & meta-analysis

**DOI:** 10.1186/1477-7517-2-4

**Published:** 2005-03-17

**Authors:** Edward J Mills, Ping Wu, Joel Gagnier, Jon O Ebbert

**Affiliations:** 1 Department of Clinical Epidemiology, Canadian College of Naturopathic Medicine, North York, Ontario, Canada; 2 Department of Clinical Epidemiology & Biostatistics, McMaster University, Hamilton, Ontario, Canada; 3 London School of Hygiene & Tropical Medicine, University of London, London, UK; 4 Institute of Medical Sciences, University of Toronto, Toronto, Ontario, Canada; 5 Department of Internal Medicine, Mayo Clinic College of Medicine, Rochester, Minnesota, USA

## Abstract

**Background:**

Acupuncture is a commonly used treatment option for the treatment of addictions such as alcohol, nicotine and drug dependence. We systematically reviewed and meta-analyzed the randomized controlled trials of acupuncture for the treatment of cocaine addiction.

**Methods:**

Two reviewers independently searched 10 databases. Unpublished studies were sought using *Clinicaltrials.gov*, the UK National Research Register and contacting content experts. Eligible studies enrolled patients with the diagnosis of cocaine dependence of any duration or severity randomly allocated to either acupuncture or sham or other control. We excluded studies of acupuncture methods and trials enrolling patients with polysubstance use or dependence. We abstracted data on study methodology and outcomes. We pooled the studies providing biochemical confirmation of cocaine abstinence.

**Results:**

Nine studies enrolling 1747 participants met inclusion criteria; 7 provided details for biochemical confirmation of cocaine abstinence. On average, trials lost 50% of enrolled participants (range 0–63%). The pooled odds ratio estimating the effect of acupuncture on cocaine abstinence at the last reported time-point was 0.76 (95% CI, 0.45 to 1.27, P = 0.30, I^2 ^= 30%, *Heterogeneity P *= 0.19).

**Conclusion:**

This systematic review and meta-analysis does not support the use of acupuncture for the treatment of cocaine dependence. However, most trials were hampered by large loss to follow up and the strength of the inference is consequently weakened.

## Introduction

Recent research on cocaine abuse and its treatment are consistent in the estimates of the social, physical, emotional and financial costs attributed to this addiction [1-5]. Currently, there is no specific pharmacologic, behavioral, or psychosocial therapy that has consistently demonstrated treatment benefits[6,7]. Most current treatment approaches are extensions of treatments ordinarily applied to alcohol or opiate addiction[8]. The limited successes in treating cocaine addiction have led patients and clinicians to examine alternative approaches.

Acupuncture is one such treatment option for addictions such as alcohol, nicotine and drug dependence[9,10]. Currently, more than 500 clinics in the United States, Canada and Europe, as well as several court-related programs, include acupuncture as a treatment option for drug dependence[11]. However, to date, the effectiveness of acupuncture as a primary treatment option or as an adjunct for cocaine dependence remains uncertain. The aim of our study was to conduct a systematic review and meta-analysis of randomized controlled trials assessing the impact of acupuncture on cocaine dependence.

## Methods

### Inclusion/Exclusion Criteria

Eligible studies enrolled patients with the diagnosis of cocaine dependence of any duration or severity randomly allocated to either acupuncture, sham or other control. Acceptable outcomes measures included: self-reported frequency of cocaine use, self-reported amount of cocaine use, or biochemical confirmation of cocaine abstinence. Biochemical confirmation of cocaine abstinence is defined as the absence of the cocaine metabolite benzoylecognine in the urine. We excluded trials of acupuncture methods and trials enrolling patients with polysubstance use or dependence.

### Literature search

Databases searched included: AMED (1985–November 2004), Campbell Collaboration (2001–January 2005), CINAHL (1982–January 2005), Cochrane Library (1998–January 2005), Cochrane Controlled Trials Registry (January 2005), E-Psyche (1993–January 2005), HTA (1988–January 2005), and MEDLINE (1966–January 2005). We additionally searched the Chinese literature through Wanfang (1997–January 2004) and the Chinese Hospital Knowledge Database (CHKD, 1994–2004). Unpublished studies were also sought using *Clinicaltrials.gov *and the UK National Research Register. We supplemented this search by hand-searching key journals and searching bibliographies of retrieved trials and reviews. We additionally contacted 5 authors to identify additional published or unpublished studies and to clarify methodological issues. There were no language restrictions.

Two reviewers (EM, PW) working independently and in duplicate, reviewed the abstracts and full text versions of identified reports and adjudicated their inclusion.

### Data extraction

Three reviewers (PW, EM, JG) working independently extracted data from the included studies using a standardized form which included: patient characteristics, treatment and control descriptions, types of outcomes measured, adverse events, and study results.

### Methodological reporting

Two reviewers (EM, PW) working independently and in duplicate assessed the reporting quality of the reports. Items collected included randomization procedure, allocation concealment, blinding of patients, care providers, and outcome assessors, adequate description of loss to follow-up, and co-interventions. These items were treated as single items and were abstracted for the purpose of sensitivity analyses in our meta-analysis, but were not used as a weighting application in our analysis.

### Statistical analyses

We measured chance-adjusted inter-rater agreement for eligibility using the kappa statistic (κ). Where reported, we extracted each trial's outcome data from the intention-to-treat analyses. If biochemical confirmation of cocaine abstinence was not reported, we contacted the authors for details. When a study had more than one control arm, we used the sham acupuncture arm as the control group. We pooled the rates of biochemically-confirmed cocaine abstinence in intervention and control groups across included trials using a random-effects meta-analysis and report the results using a forest plot describing the odds ratios (OR) and 95% confidence intervals (CI) at last reported time point. *A priori *explanations of between-study differences beyond chance included reporting of allocation concealment, use of intention-to-treat analyses, and large loss to follow-up (>50%). We tested for heterogeneity using the Zalen test and measured the proportion of between-study differences not attributable to chance with the I^2 ^statistic[12]. Our secondary analysis considered all drop outs as treatment failures. All statistics were performed using StatsDirect (Manchester, 2003).

## Results

**Figure 1:**
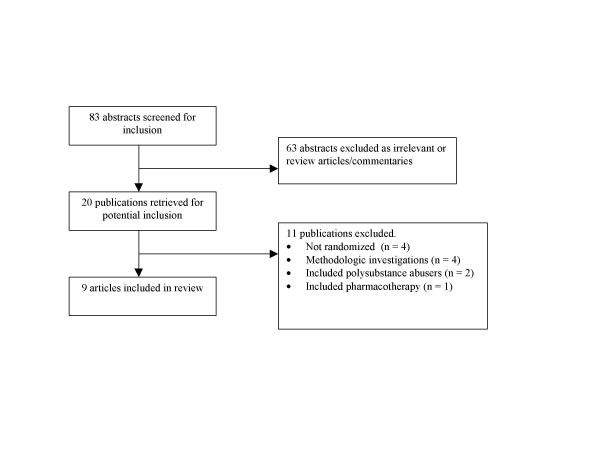
Study identification for a Systematic Review of Acupuncture for Cocaine Dependence.

The search yielded 83 relevant abstracts (Figure 1). Of these, 20 were retrieved for potential inclusion, four studies were not randomized controlled trials [13-16], four studies investigated methodological issues in acupuncture trials [17-20], 2 included polysubstance abusers[13,21] and one investigated pharmacothearapy[22]. Table 1 describes the 9 trials included in the final analysis (See additional file 1). Chance-adjusted inter-rater agreement was high (κ = 0.96, 95% CI 0.91 to 1) [23-31].

**Figure 2:**
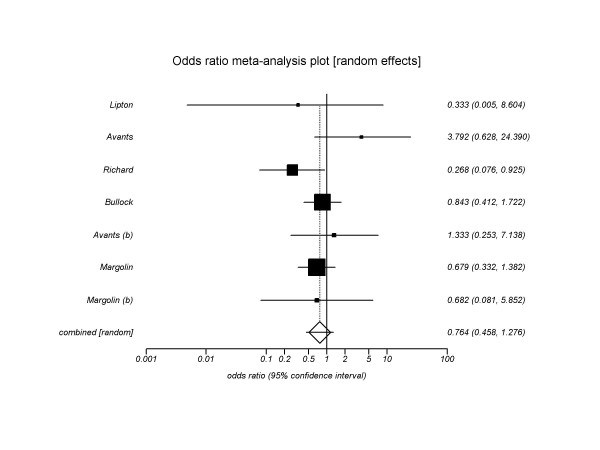
Meta-analysis of 7 trials.

### Study characteristics

The 9 RCTs were conducted in the USA and included 1747 participants: 488 participants in active groups and 821 assigned to control groups (One RCT did not describe group sizes[25]).

One RCT included only crack cocaine users[27], 5 RCTs included samples with mixed forms of cocaine abuse (eg. intravenous, inhaled, or intranasal) and 3 RCTs did not describe the type of cocaine or the route of administration[26,29,31]. RCTs enrolled participants with different rates of anti-craving medication use and 3 RCTs included only patients using methadone in addition to cocaine[23,26,31]. Three RCTs enrolled some patients using methadone[24,27,28], 2 RCTs excluded patients who used methadone[25,29] and 1 did not report the use of anti-craving medication among enrolled subjects[30].

All 9 trials employed auricular acupuncture, 4 employed a specific auricular acupuncture regimen (National Acupuncture Detoxification Association: NADA) and 2 used a combination of auricular and body points. Five trials had more than 1 control group [24-26,28,31]or randomized subjects to receive methods including relaxation[26,28,31], anti-craving medication and brainwave modification[24], or psychosocial treatment[25].

Eight trials used urine assays for cocaine metabolites (benzoylecgonine) for biochemical confirmation of abstinence at follow-up; we were able to obtain results from 7 of them. Eight trials examined the likelihood of retaining patient participation in the trial, and 5 trials examined cocaine cravings; no trials reported participant follow-up or relapse.

### Reporting quality of manuscripts

Although all trials were randomized, only 4 trials described the randomization technique[26,28,30,31]. Two trials employed restriction to balance the groups[26,28]. Allocation concealment was not adequately reported in any study. Information regarding acupuncture technique needle depth and needle type was present in 3 trials[23,26,29]. Methods of inserting a sham needle as a control were used in 8 trials[23,25-31]. Five trials reported more than 20% loss to follow-up (mean loss to follow-up across all trials was 50% [range, 0–63%])[25,26,28-30]. Only 4 trials described reasons for withdrawals[25,26,28,29].

### Meta-analysis

We pooled results from 7 trials that reported biochemical confirmation of cocaine abstinence. The pooled odds ratio estimating the effect of acupuncture on cocaine abstinence was 0.76 (95% CI 0.45–1.27, P = 0.3, I^2 ^= 30%, *Heterogeneity P *= 0.19) at the last reported time point (range 4–12 weeks) (see figure 2). Our a priori hypotheses failed to explain the heterogeneity that was present. That is, for each *a priori *explanation, the magnitude of the effect differed little irrespective of the level of the hypothesized explanatory factor. Our secondary analysis, considering all dropouts as treatment failures, resulted in a pooled odds ratio of 0.76 (95% CI, 0.54–1.08), P = 0.12, I^2 ^= 0%, *Heterogeneity P *= 0.5).

Adverse effects reported included pain and fear of needles[23,26,28,29,31] No trials reported the proportion of participants suffering adverse effects.

## Discussion

### Statement of findings

This systematic review and meta-analysis does not support the use of acupuncture for the treatment of cocaine dependence. However, most trials were hampered by large loss to follow up and the strength of the inference is consequently weakened.

### Strengths and weaknesses

We minimized publication bias by conducting an extensive search through multiple databases, including Chinese-language databases. We successfully received original data from several authors. We also sought to minimize selection and ascertainment bias by including only randomized controlled trials with biochemical confirmation of abstinence in our meta-analysis. However, large loss to follow-up and unexplained inconsistency across the included trials weakens our inferences.

The conduct of explanatory trials to ascertain the efficacy of acupuncture for cocaine addiction is challenging. We do not know whether there is a group of patients more likely to respond to this intervention who will agree to take part and stay enrolled in a clinical trial with adequate duration of follow-up. Pragmatic trials enrolling heterogeneous patients need to be large enough to detect small treatment effects and should be conducted according to the intention-to-treat principle to avoid large loss to follow-up. However, we recognize the difficulty of enrolling and maintaining patients who use or are dependent on cocaine in an RCT. Design strategies to maintain patients enrolled in trials of cocaine addiction treatment represent a research frontier.

Blinding of participants in a clinical trial is important because knowledge of group assignment may influence responses to treatment[32]. Use of sham controls and blinding of patients may prevent bias. The sham acupuncture method has been an area of debate as it is difficult to determine if a needle inserted into the skin away from designated acupuncture points is inert[20,33]. In this systematic review, 8 trials applied sham acupuncture in the control groups[23,25-31], 3 of which used the NADA technique for acupuncture protocol and these trials found inconsistent results[26,28,31]. Three trials also used a similar 5-point acupuncture protocol and did not find a significant effect[25,27,29].

We identified one additional systematic review of acupuncture for cocaine addiction which is in the process of data collection for a Cochrane review, and worked collaboratively with the primary investigator [S. Gates, personal communication]. We found systematic reviews assessing the efficacy of acupuncture in patients abusing other substances (nicotine[10], alcohol and heroin[6,9]). Our findings are consistent with these other reviews in that they included trials with large loss to follow-up and were unable to draw strong inferences about the efficacy of acupuncture as a single intervention in the management of substance abuse.

At present, no proven pharmacotherapies exist for cocaine dependence[8,34,35]. Current psychosocial strategies may only be effective in select patients with stable living conditions and social support and meta-analyses of these interventions are ongoing[6,36]. However, a lack of proof of efficacy does not indicate a lack of efficacy, and the data are dramatically hampered by dropouts. In treatment environments wishing to use multimodality therapy, acupuncture may be used for treatment of cocaine dependence since it is a relatively safe intervention and may be in line with patient values and cultural experiences. However, in resource-constricted environments, the use of acupuncture for cocaine dependence is not justified with the current evidence.

## Conclusion

Our systematic review and meta-analysis yielded inconclusive data about the effect of acupuncture. The best estimate of effect is consistent with no treatment effect. Initiatives, such as court mandated programmes, which recommend acupuncture for cocaine dependence, are not supported by the available evidence.

## Authors' contributions

Edward Mills developed the protocol, conducted the search and study selection, worked at data abstraction and quality rating and wrote the manuscript.

Ping Wu conducted the search and study selection, worked on data abstraction and quality rating, and analyzed the data.

Joel Gagnier worked at data abstraction and wrote the manuscript.

Jon Ebbert provided critical revision and writing of the manuscript.

We greatly appreciate the advice and contributions of Dr. Victor M. Montori

## Competing interests

The author(s) declare that they have no competing interests.

## Supplementary Material

Additional File 1 **Table 1. **Characteristics and findings of included studies Click here for file
