# Supplementary material for: Efficacy of acupuncture for cocaine dependence: a systematic review & meta-analysis
Source: Harm Reduct J. 2005 Mar 17;2:4. doi: 10.1186/1477-7517-2-4 (PMC1079914; doi:10.1186/1477-7517-2-4)
Supplement: Additional File 1 — Table 1. Characteristics and findings of included studies [file 1477-7517-2-4-S1.doc]

**Table 1. Characteristics and findings of included studies**

|  | ***n,* (sex), age** | **Active intervention** | **Control intervention** | **Duration & frequency of acupuncture intervention** | **Length of trial** | **Outcomes measured**  (Numbered results correspond to numbered outcomes) | **Results** | **Comments** |
| --- | --- | --- | --- | --- | --- | --- | --- | --- |
| Lipton DS  1994 [30] | n=150  (108 males, 42 females),  Age range 19-46 years | Auricular acupuncture (n=73) | - Non-specific auricular acupuncture (n=77) | 45 mins/day for 10 days | 4 weeks | 1. Daily urine assays 2. After every 4^th^ treatment asked about: cravings, if acupuncture was effective (5-pt scales) 3. Amount of crack cocaine smoked per month | 1. Cocaine abstinence at 18 days (treatment vs. sham control): OR 0.33 (95% CI: 0.005-8.60) 2. No change when heroin users excluded. 3. Five days less for both groups during treatment period. After 90 days was 5.6 for acupuncture and 3.6 for sham | - Counseling withheld during trial - Loss-to follow-up (n=93) |
| Avants SK, 1995 [23] | n=40  (22 male, 18 female),  mean age 35.2 ± 7.4 | Auricular acupuncture(n=20) | - 2mm from active group points (n=20) | 45 mins/day, 5 times/week for 6 weeks | 6 weeks | 1. Weekly urine assays for cocaine 2. Self-reported frequency of cocaine use during previous wk 3. Self-reported amount of cocaine use during previous wk 4. Cocaine craving (11 pt scale) 5. Initiation of abstinence | 1. Weekly cocaine abstinence through week 6 (treatment vs. sham control): OR 1.33 (95% CI: 0.25-7.13) 2. Cocaine use decreased from 2.1 days per week to 0.6. No differences between treatment groups. 3. Across 6 weeks self-reported cocaine use decreased from 1.5g/week to 0.2g. No differences between treatment groups. 4. Cravings decreased significantly in active group over 6 weeks. 5. 27.5% of all subjects initiated abstinence with 43.7% of the acupuncture group and 28.6% of the control needle group doing so. | - Loss-to follow-up (n=10) |
| Richard AJ,  1995 [24] | n=228  186 had usable data,  (141 male, 87 female) enrolled,  age range 18-51 years | Auricular acupuncture (n=41) and neurobehavioral therapy (NT) | - Group 1:Only NT (n=48) - Group 2: NT plus anticraving medication (n=40) - Group 3: NT plus brainwave modification (n=57) | Initially, 30 mins/day for 10 days. Followed by 30 mins/day, 3/ week.  Interested participants could continue if they wished. | 30 days | 1. Urine assays over 28 treatments 2. Self-reported frequency of use in last 30 days (10 pt scale). 3. Self-reported frequency of use in last 6 months (10 pt scale).   Self Rating Form of psychological and social functioning | 1. Cocaine abstinence at 8^th^ week (treatment vs. relaxation control): 0.26 (95% CI: 0.07-0.92) 2. Decreased in overall sample from 2.64/10 to 0.92/10. Acupuncture failed to have an effect. 3. Decrease in overall sample from 4.68/10 to 1.79/10. Acupuncture failed to have an effect. 4. Acupuncture did not show an effect. | - Loss-to follow-up (n=11) |
| Otto KC  1998 [29] | n=36  (36 males)  Mean age 38.9 years | Auricular acupuncture (n=25) | - Non-specific auricular acupuncture (n=11) | Phase I: 30-45 mins/day, 5 days/week for 2 weeks.  Phase II: 3 days/week for 2 weeks.  Phase III: 1 day/week for 24 weeks | 12weeks | 1. Urine assays(semi-weekly) 2. SCL-90 self-assessment scale 3. Hamilton depression and anxiety scales 4. Halikas cocaine craving scale 5. Halikas drug impairment rating scale | 1. No differences between treatment and control (not reported) 2. Not reported 3. No differences between treatment and control for both depression and anxiety scales 4. No differences between treatment and control 5. No differences between treatment and control   Other: Study patients as a whole received significantly more discharges than non-study patients (83% and 54% respectively: P<0.02) |  |
| Bullock ML,  1999 [25] | Study 1: n=236  Residential clients from rehabilitation centre  Study 2: n=202  Day treatment clients  Combined demographics:  (306 males, 132 females)  Mean age 30.1 | Study 1: Psychosocial Tx. plus auricular acupuncture  Study 2: Dose-response to acupuncture | - Study 1: Psychosocial Tx. alone or psychosocial Tx. plus sham acupuncture (non-specific) | Study 1: 45 min. sessions, 28 times in 8 weeks  Study 2:   - 28 treatments in 8 weeks. - 16 treatments over 8 weeks - 8 treatments over 8 weeks | 8 weeks | 1. Urine assays after 8 ^th^ week 2. Addiction severity index 3. Beck depression inventory 4. Medical outcome study (SF-36) 5. Self-administered anxiety scale 6. Preference about therapy 7. Craving measure | 1. Cocaine abstinence after 8^th^ week: (treatment vs. sham control) :0.84 (95% CI: 0.41-1.72) 2. No significant effects were found for study 1 or 2. Data is not reported. 3. No significant effects were found for study 1 or 2. Data is not reported 4. No significant effects were found for study 1 or 2. Data is not reported 5. No significant effects were found for study 1 or 2. Data is not reported 6. No significant effects were found for study 1 or 2. Data is not reported 7. No treatment by time interaction for either study 1 or 2 for the first 7days (p=0.83, p=0.91). Both studies showed a craving decrease time effect during the first 14 days of the study (p=<0.01).   No long-term differences in either study. | - High number of loss-to follow-up (n=276) - Co-interventions varied |
| Avants SK, 2000 [26] | n=82  (47 male, 35 female)  Mean age 37±6 years | NADA Auricular acupuncture (n=28) | - Group 1: Non-specific auricular acupuncture (n= 27) - Group 2: Relaxation therapy (n=27) | 40 min/day, 5/week, for 8 weeks | 8 weeks | 1. Urine assays (taken 3 times per week) 2. Addiction severity index (baseline and week 8) 3. Treatment credibility index (baseline and week 8) 4. Stages of change readiness and treatment eagerness scale (SOCRATES); baseline and week 8) 5. Ten item scale on therapeutic alliance (baseline, week 4 and 8) 6. Five measures of subjective effects of treatment sessions (each week; using a 5 pt scale) | 1. Cocaine abstinence at 8^th^ week (treatment vs. sham control): OR 3.72 (95% CI: 0.62-24.39). 2. Negative cocaine urine sample Active compared to relaxation control OR 3.41 (95% CI: 1.33-8.72), and 2.40 Active comared with needle control. 3. The acupuncture completers had significantly more cocaine-negative urine samples (7.23) than the relaxation control (2.14) or needle control (3.35) at the end of 8 weeks. 4. Therapeutic alliance scores did not differ between the two groups. 5. No difference across time or between groups 6. Relaxation controls reported significantly more relaxation after each session than the other groups (P=.004) | - Intention to treat indicates effect in active group. - Loss-to follow-up (n=20) 46% in active group, 37% in needle control and 19% in relaxation control |
| Killeen TK  2002 [27] | n=30  (18 males, 12 females)  Age range 23-45 years | NADA auricular acupuncture (n=15) | - Non-specific auricular acupuncture (n=15) | Single session of 45 minutes | 8 minutes for exposure to stimulus | 1. Cocaine craving questionnaire-now (CCQ-Now) 2. Skin conductance activity (SCA) | 1. No significant difference in craving between intervention/treatment and control (P=0.42). Significant difference between pre and post testing for entire group regardless of treatment (P<0.0001). 2. No significant difference in skin conductance between groups (P=0.64) at trial conclusion. | - Small trial - Only a single therapeutic session - No loss-to follow-up |
| Margolin A  2002 [28] | n=620  (429 males, 190 females, 1 transgendered) Mean age 38.8 | NADA auricular acupuncture(n=222) | - Group 1: Non-specific auricular acupuncture (n= 203) - Group 2: Relaxation therapy (n=195) | 40 min/day, 5/week, for 8 weeks | 8 weeks | 1. Urine assay concentration (taken 3 times per week) 2. Treatment credibility scale (pre and post-treatment) 3. Working alliance inventory 4. Measure of duration of treatment effects (each day after treatment) 5. SOCRATES (baseline) 6. Addiction severity index (baseline) | 1. Cocaine abstinence at 8^th^ week (treatment vs. sham control): 0.67 (95% CI: 0.33-1.38) 2. Treatment credibility scores were not significantly different between groups. 3. Therapeutic alliance scores were not significantly different between intervention and control(P=0.24) 4. No differences between groups. 5. Not reported 6. Severity of drug (P=0.001), psychiatric severity (P=0.001), legal problems (P=0.001), family problems (0.001) and alcohol problems (P=0.001) decreased significantly from pretreatment to post-treatment and at follow-up. There were no differences between groups. | - High number of loss-to follow-up (n=341) |
| Margolin A  2002 [31]  This study includes the analysis from Avants, 2000 (S1), as well as replicates the study and compares the study results (S2) | Study 1, n=82  This is same study as Avants 2000  Study 2, n=83  (55 males, 28 females)  Mean age 37 ± 7 | NADA auricular acupuncture  (n=36) | - Study 2: Non-specific auricular acupuncture (n=24),   Relaxation control group (n=23) | 40 min/day, 5/week, for 8 weeks | 8 weeks | 1. Urine assays taken 3 times per week 2. Self-reported cocaine use: craving scale 3. Acute affects of treatment 4. Addiction Severity Index 5. Treatment services review 6. Treatment credibility scale | 1. Cocaine abstinence at 8^th^ week (treatment vs. sham control): OR 0.68 (95% CI:0.08-5.85) 2. Rate of abstinence was higher in the acupuncture group for S1, but not for S2. 3. More relaxation in S1 participants than in S2 (P=0.015) 4. Not reported 5. More treatment satisfaction in S1 participants than in S2 (P=0.03) In S1, no difference among treatment conditions for therapeutic alliance, whereas in S2 relaxation control group showed more therapeutic alliance. 6. S2 participants found treatment significantly less credible regardless of treatment assignment than S1(p=0.003). | - Replicates the Avants, 2000. study but found inconsistent results. - Loss-to follow-up (n=19) |

ASI, Addiction Severity Index; BDI, Beck Depression Index; CCQ-Now, cocaine craving questionnaire-now; Halikas Scale; Hamilton Rating Scale for Depression and Anxiety; NADA, National Acupuncture Detoxification Association; SCL 90, Symptom Checklist 90R; SOCRATES, Stages of change readiness and treatment eagerness scale; SR, self-reported; wk, week.
